# Failure of Adaptive Self-Organized Criticality during Epileptic Seizure Attacks

**DOI:** 10.1371/journal.pcbi.1002312

**Published:** 2012-01-05

**Authors:** Christian Meisel, Alexander Storch, Susanne Hallmeyer-Elgner, Ed Bullmore, Thilo Gross

**Affiliations:** 1 Biological Physics Section, Max Planck Institute for the Physics of Complex Systems, Dresden, Germany; 2 Department of Neurology, University Clinic Carl Gustav Carus, Dresden, Germany; 3 Behavioural and Clinical Neuroscience Institute, Departments of Experimental Psychology and Psychiatry, University of Cambridge, Cambridge, United Kingdom; 4 Clinical Unit Cambridge, GlaxoSmithKline, Addenbrooke's Hospital, Cambridge, United Kingdom; University of Oxford, United Kingdom

## Abstract

Critical dynamics are assumed to be an attractive mode for normal brain functioning as information processing and computational capabilities are found to be optimal in the critical state. Recent experimental observations of neuronal activity patterns following power-law distributions, a hallmark of systems at a critical state, have led to the hypothesis that human brain dynamics could be poised at a phase transition between ordered and disordered activity. A so far unresolved question concerns the medical significance of critical brain activity and how it relates to pathological conditions. Using data from invasive electroencephalogram recordings from humans we show that during epileptic seizure attacks neuronal activity patterns deviate from the normally observed power-law distribution characterizing critical dynamics. The comparison of these observations to results from a computational model exhibiting self-organized criticality (SOC) based on adaptive networks allows further insights into the underlying dynamics. Together these results suggest that brain dynamics deviates from criticality during seizures caused by the failure of adaptive SOC.

## Author Summary

Over the recent years it has become apparent that the concept of phase transitions is not only applicable to the systems classically considered in physics. It applies to a much wider class of complex systems exhibiting phases, characterized by qualitatively different types of long-term behavior. In the critical states, which are located directly at the transition, small changes can have a large effect on the system. This and other properties of critical states prove to be advantageous for computation and memory. It is therefore suspected that also cerebral neural networks operate close to criticality. This is supported by the *in vitro* and *in vivo* measurements of power-laws of certain scaling relationships that are the hallmarks of phase transitions. While critical dynamics is arguably an attractive mode of normal brain functioning, its relation to pathological brain conditions is still unresolved. Here we show that brain dynamics deviates from a critical state during epileptic seizure attacks *in vivo*. Furthermore, insights from a computational model suggest seizures to be caused by the failure of adaptive self-organized criticality, a mechanism of self-organization to criticality based on the interplay between network dynamics and topology.

## Introduction

In the terminology of physics, a system is said to be in a critical state if it is poised on a threshold where the emergent macroscopic behavior changes qualitatively. The hypothesis that the brain is operating in such a critical state is attractive because criticality is known to bring about optimal information processing and computational capabilities [1]–[4]. Recent experimental observations of patterns of neuronal activity exhibiting scale-free distributions, a typical hallmark of phase transitions, provided further evidence for this hypothesis. Bursts of neuronal activity were first shown in reduced preparations in rat brains to follow power-law probability distributions, termed neuronal avalanches [1], [5]. More recently, neuronal avalanches were also observed in invasive recordings from monkeys and cats, strongly suggesting that criticality is a generic property of cortical network activity *in vivo* [6]–[8].

Additional evidence for the existence of a critical state in human brain dynamics comes from a recent study by Kitzbichler et al. [9]. Using magnetoencephalography (MEG) and functional magnetic resonance imaging (fMRI), the authors found power-law probability distributions of two measures of phase synchronization in brain networks. As confirmed by computational models, these distributions show power-law scaling specifically when those model systems are in a critical state resulting in strong evidence that human brain functional systems exist in an endogenous state of dynamical criticality at the transition between an ordered and a disordered phase.

Theory predicts local events to percolate through the system in the form of avalanches of activity at the critical state [10]. Such a critical state requires a homeostatic regulation of activity leading to a balance of excitation and inhibition in order to prevent states where events are either small and local or very large, engaging most of the network. A promising mechanism showing robust self-organized criticality (SOC) - the ability of systems to self-tune their operating parameters to the critical state - came from the discovery of network-based mechanisms, which were first reported in [11] and explained in detail in [12], [13]. These works showed that *adaptive networks*, i.e., networks that combine topological evolution of the network with dynamics in the network nodes [14], can exhibit highly robust SOC based on simple local rules. In computational models it could be shown that realistic local mechanisms based on this adaptive interplay between network activity and topology are sufficient to self-organize neuron networks to a critical state, providing a plausible explanation of how criticality in the brain can be achieved and sustained [15]–[17].

A so far unresolved question concerns the medical relevance of critical brain activity. Diseases in the central nervous system are often associated with altered brain dynamics. It has been hypothesized that the dynamical properties characterizing a critical state may be seen as an important marker of brain well-being in both health and disease [18]. Epilepsy is a malfunction of the brain associated with abnormal synchronized firing of neurons during a seizure [19]. The increased collective neuronal firing during attacks has been speculated to be linked to a pathological deviation away from a critical state [20]. Evidence supporting this idea comes from recent *in vitro* studies of animal brains. There, application of receptor blockers could drive network dynamics away from its normal state where activity patterns of neuron dynamics deviated from a power-law [6], [21].

Here, we confirm the previously observed power-law distribution of phase-lock intervals (PLI) with a complementary experimental methodology, providing additional evidence for the criticality hypothesis. Furthermore, we present evidence that human brain networks *in vivo* are not in a critical state during epileptic seizure attacks. Deriving the distribution of PLI from electrocorticogram (ECoG) data as an indicator of critical brain dynamics as proposed in [9], we find that the system deviates from scale-free behavior during seizures. Combined with results from a computational model exhibiting SOC these observations suggest that dynamics of brain networks is typically close to criticality, but departs from the critical state during epileptic seizures. Together these results hint to the failure of adaptive SOC as a cause for seizure generation.

## Results

### Analysis of ECoG Data

We investigated data sets from ECoG acquired during presurgical monitoring of patients suffering from focal epilepsy. Data were continuously sampled at 200 Hz (patients 1–7) or 256 Hz (patient 8) with the number of channels ranging from 30 to 45 for different patients. The time series recorded from the anatomical site where the epileptic focus was assumed typically included one or more neurographically-identifiable seizure attacks.

To test brain dynamics for signatures of criticality we analyzed ECoG activity in different time windows. The data sets were split in intervals of 150 seconds length (30000 sample steps at 200 Hz sampling, 38400 in the case of 256 Hz) with consecutive intervals overlapping by 100 seconds (20000 sample steps at 200 Hz, 25600 at 256 Hz). Following the approach in [9], we determined the distribution of phase-locking intervals (PLI) as an experimentally accessible indicator of critical brain activity. The length of time windows was chosen to be long enough to give a reliable estimate of the distribution of PLI on the one hand and allow observation of its evolution in time on the other hand. For each of these sets, we calculated phase-lock intervals and determined their cumulative density distributions for scales 2, 3 and 4 corresponding to frequency intervals 50–25 Hz, 25–12.5 Hz and 12–6 Hz for patients 1–7 (P1–P7) and 64–32 Hz, 32–16 Hz, 16–8 Hz for patient 8.

The distributions for all scales closely follow a power-law probability distribution with 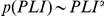 during pre-ictal time intervals and 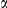 between −2 and −3.5. Statistical tests based on the Kolmogorov-Smirnov statistic and likelihood ratios [22] showed that the hypothesis of a power-law PLI distribution could not be rejected for most pre-ictal data sets, furthermore a recent comprehensive analysis of various fitting functions applied to PLI distributions had revealed a power-law to be the most likely fit [9]. The apparent robustness of the power-law against exact conditions (different anatomical regions with varying number of channels) strengthens the hypothesis of the relevance of a critical state in human brain dynamics.

**Figure 1:**
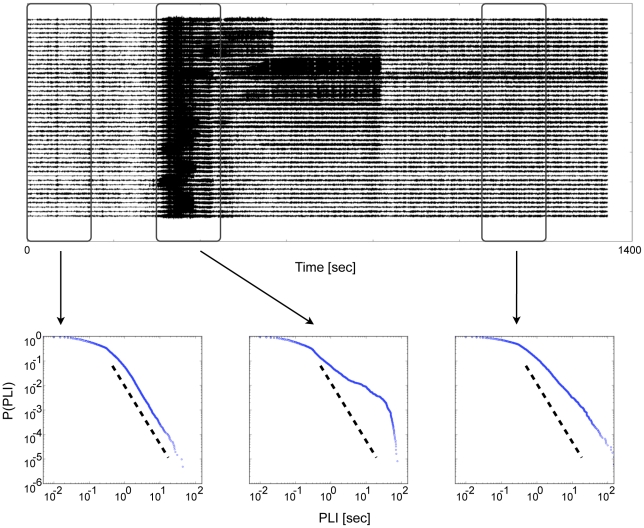
The distribution of phase-locking intervals deviates from a power-law during epileptic seizures. Top: The electrocorticogram (ECoG) recording shows the onset of a focal epileptic seizure attack around 300 seconds time. Bottom: Cumulative distributions of phase-locking intervals (PLI) are obtained during three time intervals of 150 seconds: pre-ictal (left), ictal (middle) and post-ictal (right). Dashed lines indicate a power-law with exponent −3.1. While the distribution appears to follow a power-law during the pre-ictal period, intervals of increased phase-locking disturb this characteristic distribution with the onset of seizure activity. Data shown are from patient 1 at scale 3, corresponding to the frequency band 25–12.5 Hz.

**Figure 2:**
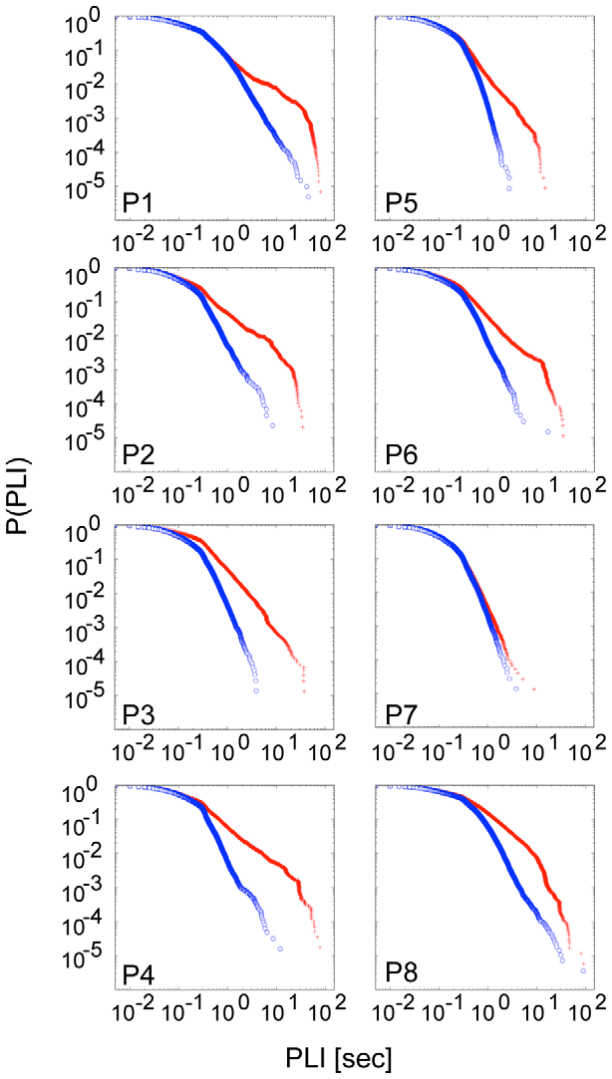
Comparison of PLI distributions derived from the first (pre-ictal) time interval (blue curve) and an interval during the seizure attack (red curve). Distributions from seizure intervals tend to exhibit longer periods of phase-locking resulting in a deviation from a power-law of the distribution's tail. Plots are shown for scale 3 corresponding to the frequency band 25-12.5 Hz for patients 1–7 (P1–P7) and 32-16 Hz for patient 8 (P8), respectively.

While the PLI distribution followed a power-law in time intervals preceding the seizure onset, a deviation from power-law behavior was observed in intervals containing the seizure attack. Figure 1 shows distributions of PLI derived from a pre-ictal, an ictal and a post-ictal time interval. The probability to find longer PLI increased during attacks thereby destroying the scale-free property of the original distribution. After the seizure this distribution slowly relaxed back to a power-law. In Figure 1 this relaxation is not yet complete in the post-ictal time interval as there is still some residual seizure dynamics in the ECoG recording. The qualitative change away from a power-law distribution during seizures could be observed in all 8 patients and across scales (Figure 2). Distributions for all consecutive time windows and all scales from patient 1 can be found in the supplementary material (Figure S1).

A more quantitative estimate of the deviation from the pre-ictal state can be obtained by calculating 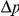, a measure previously proposed to characterize the divergence from a critical state [17]. This measure captures the deviation from a given empirical distribution from a power-law. The power-law fitted to the first (pre-ictal) interval was thereby taken as a reference and subtracted from the cumulative PLI distributions of subsequent time intervals.

**Figure 3:**
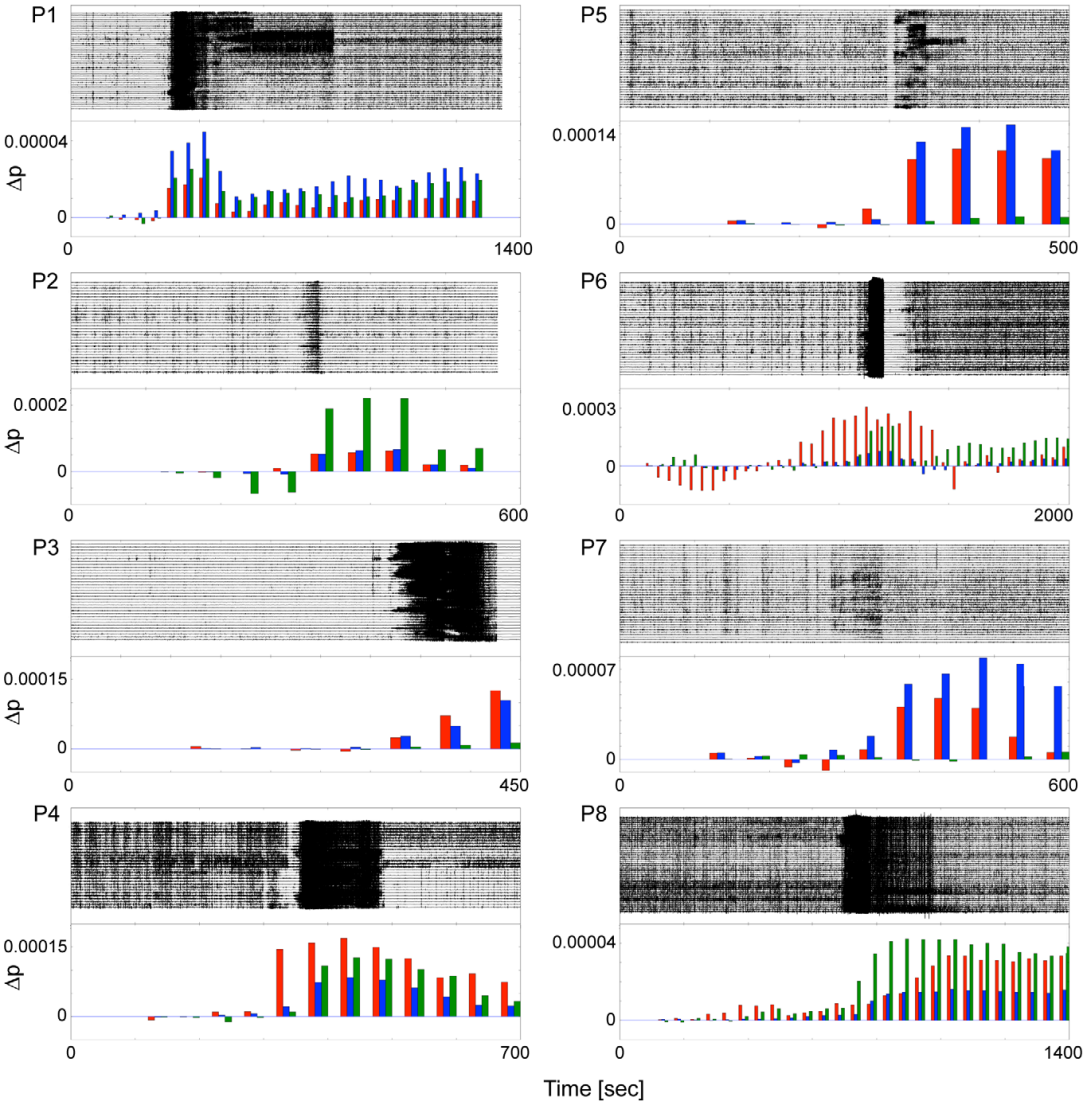
Development of the deviation from a power-law. ECoG recordings from 8 patients showing a focal seizure attack are shown along with 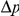 values for consecutive time windows of 150 seconds duration overlapping by 100 seconds. The power-law fit of data in the first time window was taken as the reference to calculate 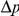. Although different in extent, an increase of 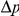 quantifying the deviation from the initial pre-ictal distribution can be observed during seizures for all patients and different scales (scale 2 red, scale 3 blue, scale 4 green).

During time intervals preceding the seizure 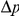 stayed at low values indicating no significant deviation from a power-law. In time windows containing seizure activity, 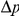 increased to positive values, which is in agreement with the qualitative assessment from visual inspection showing a divergence from the initial distribution. After seizure attacks, a slow decrease of 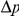 could be observed suggestive of a relaxation process back toward a power-law distribution (Figure 3).

### Computational Model

For obtaining further insights into the underlying dynamics of the power-law probability distribution of PLI and its absence during epileptic seizure attacks, we compared experimental results to a simple computational model exhibiting self-organized criticality. Our numerical results build on a model proposed by Bornholdt and Rohlf [12]. This model robustly self-organizes toward a critical state and is sufficiently simple to allow for an understanding of the underlying mechanism by which this self-tuning is accomplished. Specifically, the adaptive interplay of network dynamics and topology, a mechanism also at work in more elaborate models of SOC in neural networks [15]–[17], robustly organizes systems parameters, in this case the average connectivity 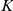, toward values 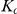 where the network's state is at a phase transition between ordered and disordered dynamics.

**Figure 4:**
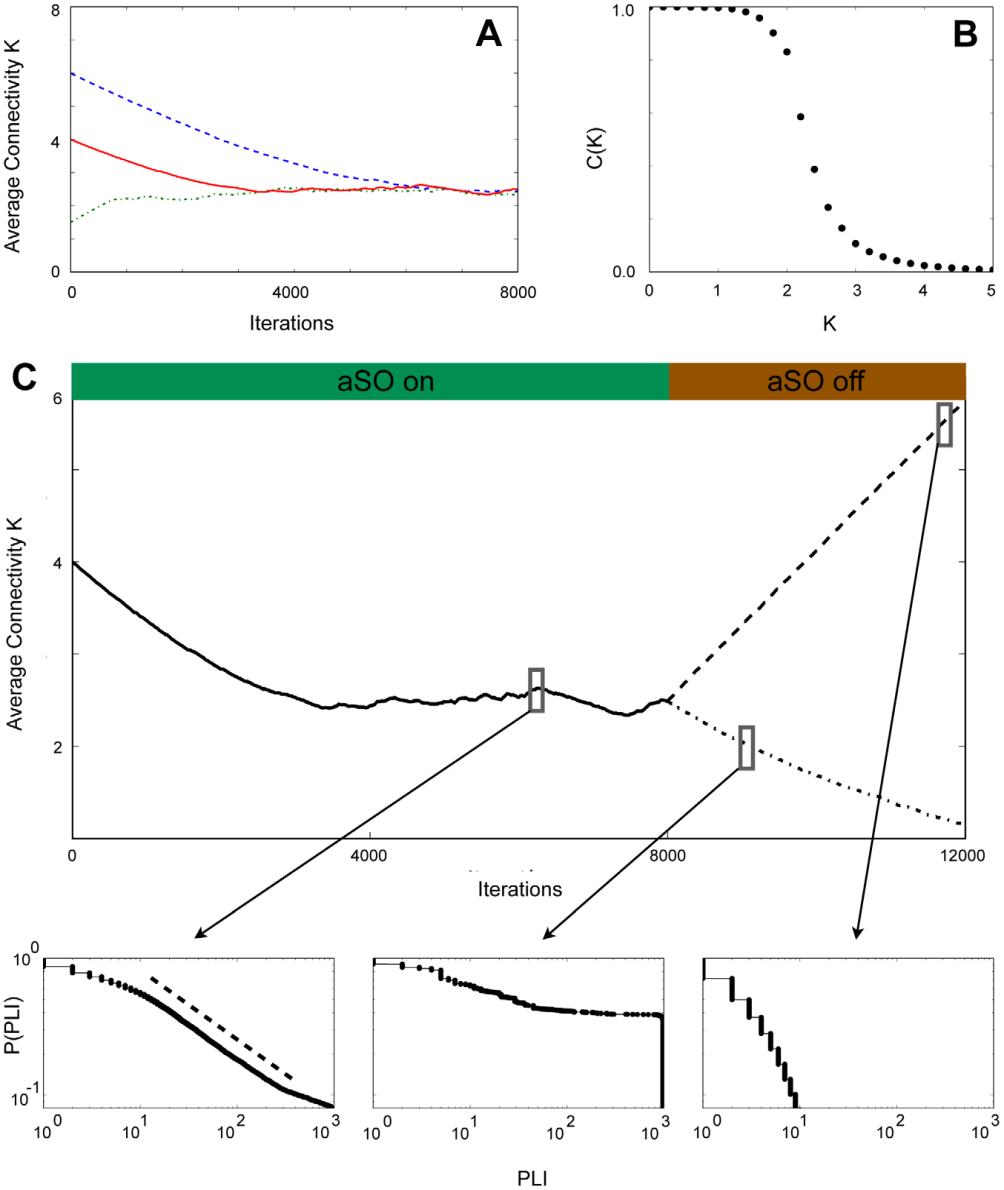
Distribution of PLI in a model exhibiting self-organized criticality. A Through an adaptive interplay of network dynamics and topology, the Bornholdt model self-organizes toward a characteristic connectivity independent of initial conditions. The plot shows the evolution to a characteristic connectivity of approximately 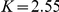 in a network of 1024 nodes for three different initial connectivities, 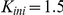, 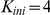 and 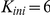. B At this self-organized connectivity the network exhibits a phase transition between order and disorder. The plot shows the frozen component 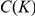 defined as the fraction of nodes that do not change their state along the attractor as a function of networks' average connectivities 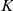 for a network of 1024 nodes. The data were measured along the dynamical attractor reached by the system, averaged over 100 random topologies for each value of 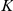. A transition around a value 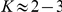 can be observed. C After a period of self-organization based on the adaptive interplay between topology and dynamics (aSO on, full black line), links were added and deleted solely with a certain probability independent of node activity (aSO off, dashed line: links were added with 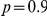 and deleted with 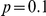, point-dashed line: links added with 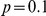, deleted with 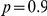). Each iteration marks a topological update of the network, between iterations network activity was limited to 1000 time steps where topology was not changed. Phase-lock intervals between 20 randomly chosen nodes were calculated for scale 1 from 100 consecutive iterations at three time points: at the self-organized connectivity (bottom left), at a connectivity lower (bottom middle) and higher (bottom right) than the evolved connectivity. The distribution of PLI follows a power-law only at the self-organized connectivity (bottom left). All depicted distributions are cumulative distributions. The dashed line marks a power-law with exponent −1.5 to guide the eye.

For a network with 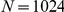 nodes adaptive self-organization (aSO) leads the average connectivity to settle at values around 2.55 independent of initial conditions (Figure 4A). The frozen component 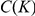 defined as the fraction of nodes that do not change their state along the attractor undergoes a transition at this self-organized connectivity (Figure 4B). In the large system size limit 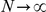 the networks evolve to a critical connectivity 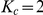 where the transition from the frozen to the chaotic phase becomes a sharp step function [12]. The system therefore exhibits a phase-transition with a frozen/ordered phase at lower connectivities and a disordered phase of network dynamics at higher connectivities.

Our goal was to compare the distribution of PLI at the self-organized connectivity and at connectivities below and above it. This would correspond to critical dynamics as well as dynamics in the ordered/frozen and disordered phase respectively. We therefore let the network evolve according to the adaptive self-organization (aSO) process described in [12] and the methods section (iterations 0–8000) and derived PLI of 100 consecutive iterations at some point when the average connectivity had settled around 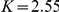. There, the distribution of PLI appeared to follow a power-law (Figure 4C). More precisely, statistical tests [22] revealed that the hypothesis of a power-law for the distribution of PLI cannot be rejected at the self-organized connectivity.

Next, we switched the aSO off at 8000 iterations, instead adding and deleting links with a certain probability independent of node activity after this point (iterations 8001–12000). We considered two cases: First, where links were added with probability 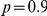 and deleted with 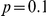 and second, where links were added with 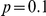 and deleted with 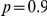 after each iteration following the first 8000 iterations. In the first case more links were effectively added so that the average connectivity organized to higher values. The second case led to a net decrease in links resulting in a lower average connectivity (Figure 4C). We again derived PLI of 100 consecutive iterations at some time for each of the two cases. In both cases the distribution of PLI deviated from a power-law consistent with a state away from critical dynamics (Figure 4C). The distribution at connectivities corresponding to the ordered phase of network dynamics is shifted towards larger PLI similar to the one observed during epileptic seizure attacks (bottom right in Figure 4C).

The close agreement between patient and model data suggests that the deviation from a power-law observed during epileptic seizure attacks indicates a shift of dynamics toward an ordered phase. In the model above this corresponds to the phase of frozen dynamics. It further hints that it is the mechanism of adaptive SOC, the ability to tune system parameters to values where network dynamics is at a phase transition and PLI are distributed according to a power-law, that could fail during epileptic seizure attacks in neuron networks in the brain.

## Discussion

The relevance of critical brain dynamics is currently a heavily debated topic. Indirect evidence for such a state comes from power-law distributed observables in neurophysiological data. Power-laws can arise through various mechanisms such as the combination of two exponential distributions or random extremal processes such as the Omori law for earthquake aftershocks for example [23]. With respect to neural dynamics power-law behavior can be generated by filtering properties of neural tissue [24]. Although various mechanisms can result in an event size distributions exhibiting power-laws [25], such distributions also arise when a system is in a critical state [10]. The observation of power-laws therefore provides an indication but not a proof of critical dynamics. Conversely, the absence of power-law scaling would provide a strong evidence against criticality.

The power-laws observed in neural data are consistent with the hypothesis of neural criticality. The hypothesis is further supported by a) evolutionary arguments highlighting the advantages of operating in a critical state [26] and b) the formulation of fairly realistic models [13], [15]–[17] explaining how a critical state can be reached as a result of well-known neural and synaptic mechanisms. Comparison of experimental data to data from a computational models known to exhibit critical dynamics can provide support for the conclusion that an experimentally observed power-law is a signature for critical dynamics.

Recently, the power-law distribution of phase-lock intervals between pairs of neurophysiological time series was shown to be a specific hallmark of dynamic criticality in human brain dynamics [9]. Following the line of arguments outlined above, the authors demonstrated power-law scaling of PLI both in neurophysiological data and also in Ising and Kuramoto model when these systems were tuned to a phase transition. In this work we extend these computational results to a third model known to exhibit SOC [12]. Together these numerical results provide strong support that the observed power-law distribution of PLI is characteristic for a system at a phase transition between ordered and disordered dynamics.

Using this indicator on ECoG data, a complementary experimental methodology to [9], we confirm the previously observed power-law distribution of PLI, providing additional evidence for the criticality hypothesis. Secondly, we show that the critical state is disturbed during epileptic seizure attacks. More precisely, the distribution of the PLI synchronization measure deviates from a power-law, characterizing the critical state of normal neuronal dynamics, during epileptic seizures, providing the first direct evidence of disturbed critical dynamics related to a pathology *in vivo*.

Our findings support the notion of a physiological default state of balanced brain dynamics between regimes of exuberant and frozen activity. Physiological neuronal activity is characterized by intermittent periods of synchronization between different anatomical regions. In terms of dynamical system's theory, such a state corresponds to a critical state at a phase transition between order and disorder. A deviation from this balanced state toward dynamics with pathologically increased times of synchronous activity as observed in epileptic patients leads to a deviation from the physiological critical state resulting in impaired functionality.

Optimal information processing capabilities of neuron networks have been related to a critical state before [1], [26], [27]. The requirement for such functional capabilities could be differently pronounced in different brain networks and at different times. From this perspective, it is very unlikely that all regions in the brain are tuned to a critical state at all times. Our results in fact show that the goodness of the power-law in the PLI distribution varies between different regions and times (see for example the rather poor power law of P7 in Figure 2). One could speculate that self-organization to a critical state is differently pronounced in distinct anatomical regions perhaps dependent on distinct functional requirements.

A mechanism by which complex networks can self-organize toward a critical state is based on the adaptive interplay between the dynamics *on* the network, i.e. neuronal activity, and the dynamics *of* the network, i.e. the shaping of synaptic connections. Through this interaction system parameters can be locally tuned to a state of global criticality [12], [13]. While the simple model described in this work captures these essential ingredients allowing for an understanding of the underlying concept, more elaborate mechanism can be expected to be at work in real-world neuron networks [15]–[17], [28]. It is conceivable that physiological neuron networks in the brain tune their parameters to more than one parameter to reach a state of criticality. Besides the average connectivity of the network 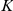, the balance between excitation and inhibition, for example, has been shown to be an important parameter to sustain a homeostatically balanced critical state and prevent regimes of overly synchronized activity. The robust mechanism of adaptive SOC allows neuron networks in the brain to maintain close to a critical state characterized by dynamics exhibiting power-law probability distributions even while network dynamics and topology undergo changes.

Along this line of arguments the deviation from a power-law distribution of PLI reported here can be interpreted as a shift away from a balanced critical state and to our knowledge constitutes the first proof of impaired critical dynamics related to a pathology *in vivo*. This observation is supported by experimental results from *in vitro* studies. The application of receptor blockers in slice preparation of animal brains resulting in an excess of excitation in the network destroyed the power-law distributed avalanches of neuronal activity and led to increased avalanche sizes corresponding to a super-critical state [5], [6]. Analogously, human tissue removed from epilepsy patients exhibited abnormally regulated avalanches with periods of hyperactivity [29].

In summary, experimental results from *in vitro* experiments [5], [6] and *in vivo* observations presented here combined with insights from computational models based on adaptive SOC [12], [15]–[17], [28] suggest the failure of the adaptive interplay between neuron activity and network topology to lead to the deviation from a critical state. There pathological, in the case of epilepsy overly synchronized, activity patterns are observed. A deviation from the default critical state towards a dynamical regime with decreased phase-locking is also conceivable. For instance in neurodegenerative diseases with impaired neuronal connectivity, the deviation from a power-law of PLI could potentially be used to identify and characterize these pathological conditions.

## Materials and Methods

### Acquisition and Preprocessing of Experimental Data

Eight patients undergoing surgical treatment for intractable epilepsy participated in the study. Patients underwent a craniotomy for subdural placement of electrode grids and strips followed by continuous video and ECoG monitoring to localize epileptogenic zones. Solely clinical considerations determined the placement of electrodes and the duration of monitoring. Positions of the electrodes from patients 1–7 can be found in the supplementary material (Figure S2). All patients provided informed consent. ECoG signals were recorded by the clinical EEG system (epas 128, Natus Medical Incorporated) and bandpass filtered between 0.53 Hz and 70 Hz. Data were continuously sampled at a frequency of 200 Hz (patients 1–7) and 256 Hz (patient 8, [30]). The study protocols were approved by the Ethics Committee of the Technical University Dresden.

### Estimation of Phase Synchronization

To derive a scale-dependent estimate of the phase difference between two time series, we follow the approach described in ref. [9] using Hilbert transform derived pairs of wavelet coefficients [31]. We define the instantaneous complex phase vector for two signals 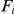 and 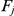 as:
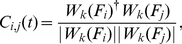
(1)where 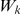 denotes the 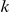-th scale of a Hilbert wavelet transform and 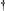 its complex conjugate. A local mean phase difference in the frequency interval defined by the 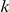-th wavelet scale is then given by
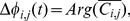
(2)with
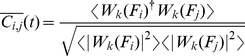
(3)being a less noisy estimate of 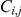 averaged over a brief period of time 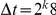 [9]. Intervals of phase-locking can then be identified as periods when 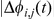 is smaller than some arbitrary threshold which we set to 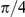 here. We also require the modulus squared of the complex time average, 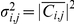, to be greater than 0.5, limiting the analysis to phase difference estimates above this level of significance.

### Estimation of Deviation from a Power-law Distribution

To quantify the deviation from a power-law we defined a measure 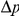 similar to ref. [17]. 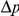 measures the difference between the cumulative density distribution of phase-lock intervals and a theoretical power-law distribution 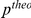 obtained from a fit of the experimental data [22]. 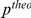 is calculated from the first time-interval (0–150 seconds) of a data set. For each time-interval of 150 seconds duration, 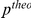 is then subtracted from the cumulative density distribution of PLI, 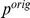, for each data point corresponding to a phase-lock interval 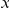 and normalized by the number of data points 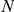:
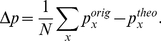
(4)


Positive values of 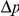 indicate a deviation with increased intervals of phase-locking, negative values indicate decreased phase-locking compared to the reference power-law distribution.

### Computational Model

An influential model explaining how dynamical systems can self-organize towards a critical state was introduced in ref. [12]. The mechanism is based on the adaptive interplay between the dynamics of the nodes in the network (dynamics on the network) and the rewiring of the network's topology (dynamics of the network). More precisely, the topology of the network is changed according to the activity of the nodes in the network so that on average active nodes lose links and frozen nodes grow links. This local rewiring leads to a robust evolution towards a critical connectivity 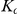 where the system is at a phase transition between order and disorder [12].

We first instantiated this model in a network of 1024 randomly interconnected binary elements with states 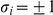 which are updated in parallel and scanned for local rewiring of connections. After 1000 time steps the network's topology was updated by picking one random node and either adding/deleting an incoming link to it depending on its activity during the last 1000 time steps [12]. This process was iterated many time leading the network's topology to evolve towards a critical connectivity of 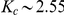. Our objective was to compare the distribution of phase-lock intervals between the activity of pairs of nodes for different average connectivities to provide a reference for comparable analysis of neurophysiological time series. We therefore monitored states 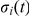 of 20 randomly chosen nodes in a network after it had self-organized to the critical connectivity and derived the distribution of PLI.

To organize the network away from 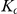 we added and deleted links solely based on probability, independent of node activity after 8000 iterations. Dependent on the probalitiy with which links were added/deleted the average connectivity organized to higher/lower values at which we again monitored the states 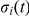 of 20 randomly chosen nodes and derived the distribution of PLI. We found that the probability distribution of phase-lock intervals demonstrated power-scaling specifically when the system was at the self-organized critical connectivity whereas distributions at lower/higher connectivities deviated from a power-law showing periods of increased/decreased phase-locking.

## Supporting Information

Figure S1 Cumulative distribution of phase-lock intervals for consecutive time windows and different scales (scale 2 green, scale 3 red, scale 4 blue) from patient 1.
(TIFF)

Figure S2 Schematic drawings of the positions of the electrodes from patients 1 to 7.
(TIFF)
