# Supplementary figures and images for: Failure of Adaptive Self-Organized Criticality during Epileptic Seizure Attacks
Source: PLoS Comput Biol. 2012 Jan 5;8(1):e1002312. doi: 10.1371/journal.pcbi.1002312 (PMC3252275; doi:10.1371/journal.pcbi.1002312)

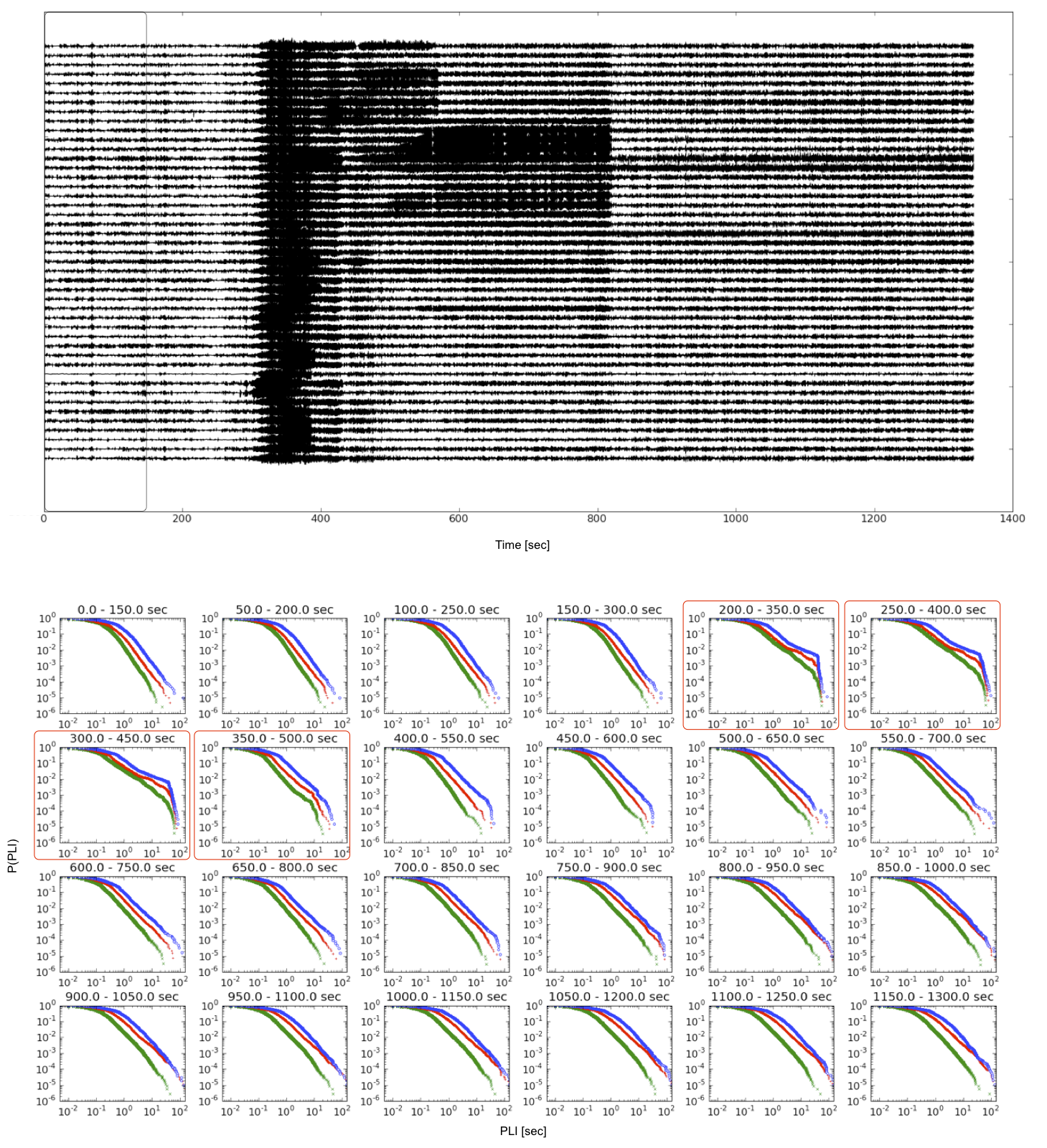

Supplement: Figure S1 — Cumulative distribution of phase-lock intervals for consecutive time windows and different scales (scale 2 green, scale 3 red, scale 4 blue) from patient 1. (TIFF) [file pcbi.1002312.s001.tiff]

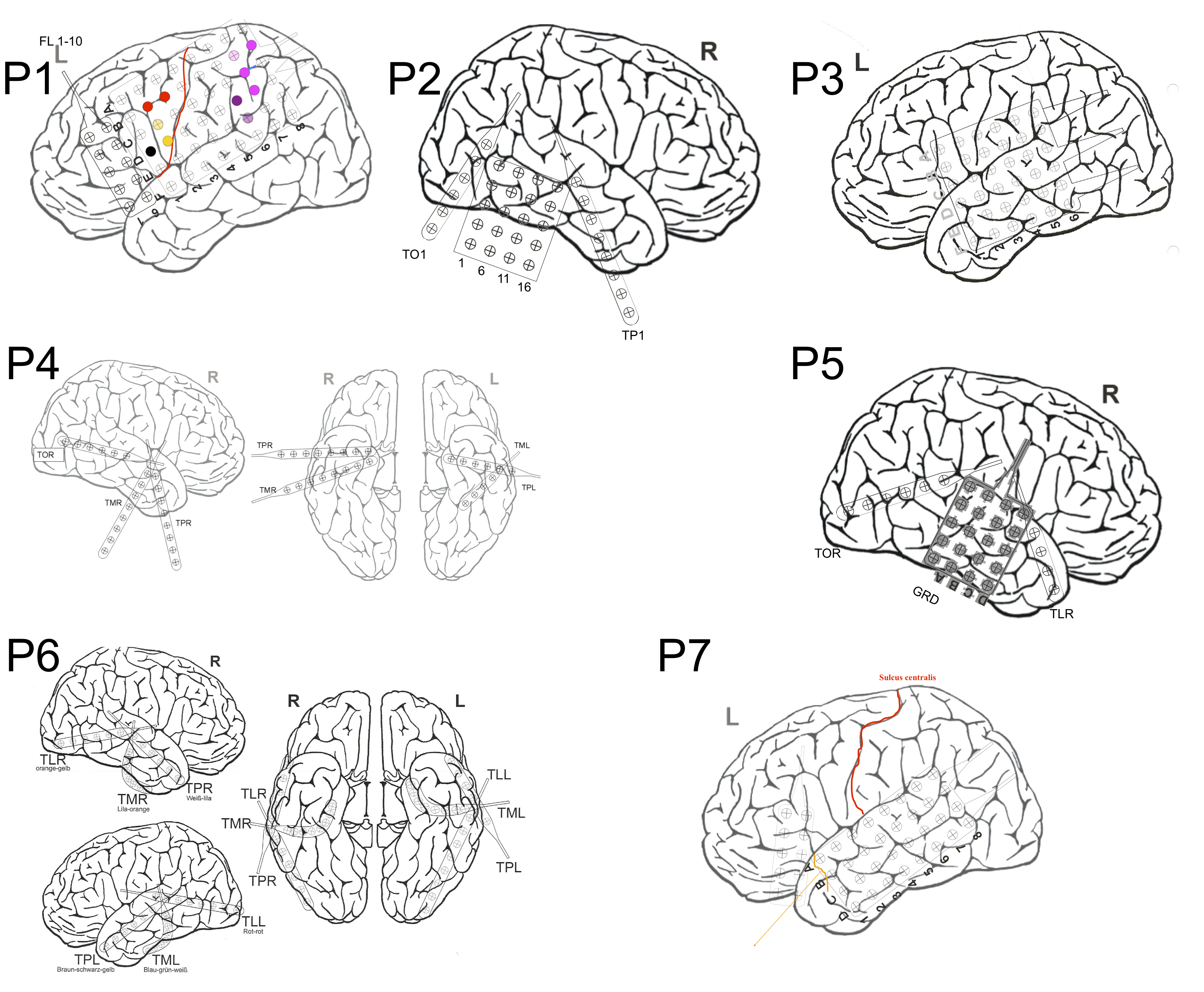

Supplement: Figure S2 — Schematic drawings of the positions of the electrodes from patients 1 to 7. (TIFF) [file pcbi.1002312.s002.tiff]
